# Effects of normobaric oxygen and melatonin on reperfusion injury: role of cerebral microcirculation

**DOI:** 10.18632/oncotarget.5773

**Published:** 2015-09-21

**Authors:** Mustafa C. Beker, Ahmet B. Caglayan, Taha Kelestemur, Berrak Caglayan, Esra Yalcin, Burak Yulug, Ulkan Kilic, Dirk M. Hermann, Ertugrul Kilic

**Affiliations:** ^1^ Department of Physiology and Regenerative and Restorative Medical Research Center, Istanbul Medipol University, Istanbul, Turkey; ^2^ Department of Neurology, Istanbul Medipol University, Istanbul, Turkey; ^3^ Department of Neurology, University Hospital Essen, Essen, Germany

**Keywords:** hyperoxia, reperfusion injury, cerebral microcirculation, BBB permeability, NOS, Pathology Section

## Abstract

In order to protect the brain before an irreversible injury occurs, penumbral oxygenation is the primary goal of current acute ischemic stroke treatment. However, hyperoxia treatment remains controversial due to the risk of free radical generation and vasoconstriction. Melatonin is a highly potent free radical scavenger that protects against ischemic stroke. Considering its anti-oxidant activity, we hypothesized that melatonin may augment the survival-promoting action of normobaric oxygen (NBO) and prevent brain infarction. Herein, we exposed mice to 30 or 90 min of intraluminal middle cerebral artery occlusion (MCAo) and evaluated the effects of NBO (70% or 100% over 90 min), administered either alone or in combination with melatonin (4 mg/kg, i.p.), on disseminate neuronal injury, neurological deficits, infarct volume, blood-brain barrier (BBB) permeability, cerebral blood flow (CBF) and cell signaling. Both NBO and particularly melatonin alone reduced neuronal injury, neurological deficits, infarct volume and BBB permeability, and increased post-ischemic CBF, evaluated by laser speckle imaging (LSI). They also improved CBF significantly in the ischemic- core and penumbra, which was associated with reduced IgG extravasation, DNA fragmentation, infarct volume, brain swelling and neurological scores. Levels of phosphorylated Akt, anti-apoptotic Bcl-xL, pro-apoptotic Bax and endothelial nitric oxide synthase (NOS) were re-regulated after combined oxygen and melatonin delivery, whereas neuronal and inducible NOS, which were increased by oxygen treatment, were not influenced by melatonin. Our present data suggest that melatonin and NBO are promising approaches for the treatment of acute-ischemic stroke, which encourage proof-of-concept studies in human stroke patients.

## INTRODUCTION

Rapid post-ischemic reperfusion, initiated either by tissue-plaminogen activator-induced thrombolysis or interventional clot removal, is critical for successful neurological recovery following ischemic stroke [1]. Rapid reperfusion is a *sine qua non* condition for post-ischemic tissue survival, while in non-reperfused tissue or in tissue that is reperfused with delay, depending on the duration of ischemia secondary brain damage may result in disseminate neuronal death or brain infarction [2, 3]. A major contributor to ischemic reperfusion injury is reactive oxygen species (ROS), which are formed in response to tissue re-oxygenation and promote inflammatory responses that activate neuronal apoptosis [4].

Exposure to normobaric oxygen (NBO) is a simple, inexpensive and easy-to-access strategy that allows to enhance tissue re-oxygenation even in the hands of paramedics in ischemic stroke. Studies in rats using electron paramagnetic resonance-guided oxymetry have shown that penumbral partial oxygen pressure (pO_2_), which decreases to ≈30% of pre-ischemic levels after middle cerebral artery occlusion (MCAo), is doubled when 95% oxygen is administered in the ischemic phase [5].

As a consequence of enhanced tissue oxygenation, NBO exposure during ischemia reduced ischemic brain injury, improved neurological function, decreased cerebral ROS formation and diminished matrix metalloproteinase and initiator caspase-8 activation in ischemic brain tissue [5–8]. In contrast, NBO treatment during reperfusion had no beneficial effect or less significant effect as compared with NBO treated animals during ischemia [5, 9]. These findings were interpreted such that ROS formation, triggers lipid peroxidation and cell membrane damage, and consequently neuronal death [10].

Melatonin is a potent free radical scavenger with excellent blood-brain barrier (BBB) permeability [10–12], which protects against focal cerebral ischemia in the mouse [13–17] and rat [18–20] by increasing phosphorylated Akt, increasing anti-apoptotic Bcl-xL, decreasing pro-apoptotic Bax, decreasing neuronal NO synthase (nNOS), decreasing inducible NO synthase (iNOS), increasing endothelial NO synthase (eNOS) and decreasing activated caspase-3 proteins.

The combined free radical scavenging and neuroprotective properties render melatonin particularly suitable for ischemic stroke treatment; supporting furthermore the hypothesis that melatonin might reverse possible detrimental effects of NBO after focal cerebral ischemia. Moreover, the effect of NBO on reperfusion injury and its underlying mechanism, which is an important concern of clinical studies, is not well studied. To investigate these, we herein exposed mice to focal cerebral ischemia induced by 30 or 90 min MCAo, evaluated the effects of 21, 70 or 100% NBO alone and in combination with vehicle or melatonin on i) disseminate neuronal injury assessed by terminal transferase dUTP nick end labeling (TUNEL), ii) neurological deficits, infarct volume, brain swelling and blood-brain barrier (BBB) permeability, iii) real time semi-quantitative cerebral blood flow (CBF) examined by laser speckle imaging (LSI), and iv) the abundance of phosphorylated Akt, Bcl-xL, Bax, nNOS, iNOS and eNOS proteins in ischemic brain tissue.

## RESULTS

### Effects of NBO and melatonin on disseminate neuronal injury

**Figure 1:**
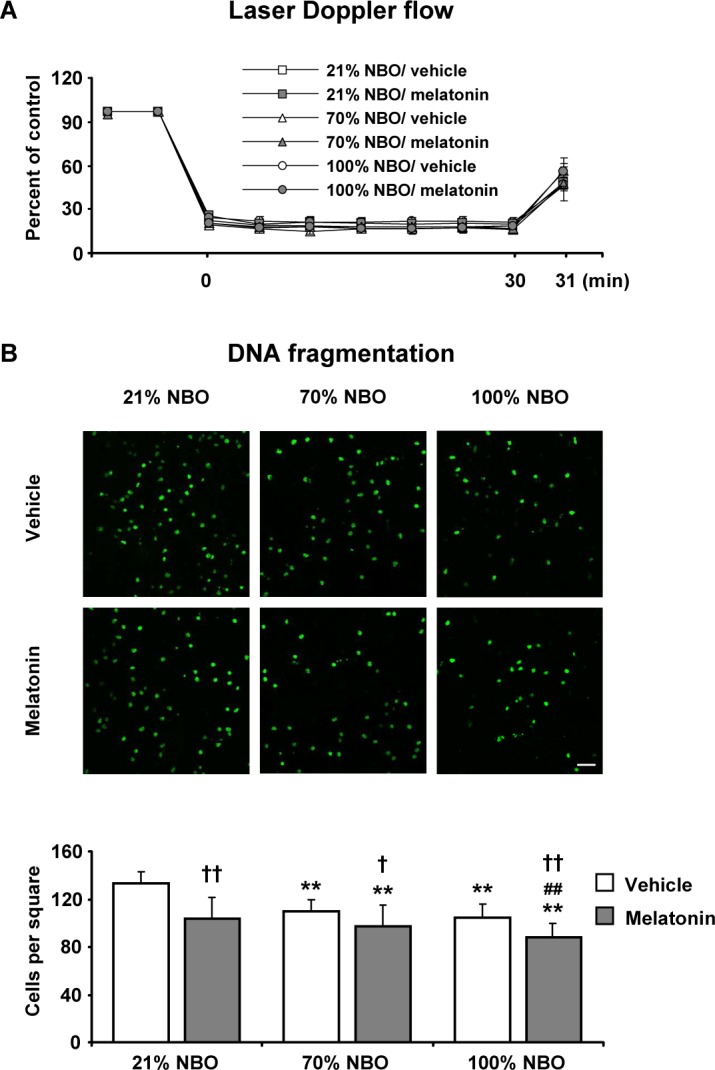
Effects of normobaric oxygen and melatonin on disseminate neuronal injury **A.** Laser Doppler flow (LDF) recordings during ischemia and initial reperfusion and **B.** disseminate neuronal injury in the striatum assessed by terminal transferase dUTP nick end labeling (TUNEL) in mice submitted to 30 min of intraluminal middle cerebral artery occlusion (MCAo), which induces disseminate neuronal injury in the striatum [3, 21]. During the first 90 min of reperfusion, mice had been exposed to 21% (ambient air), 70% and 100% normobaric oxygen (NBO). Vehicle or melatonin (4 mg/kg) was intraperitoneally administered after reperfusion onset, and mice were sacrificed 72 hours later. Note that the number of TUNEL+ (i.e., DNA-fragmented) cells is significantly decreased by NBO alone and melatonin alone and furthermore synergistically reduced by combined NBO and melatonin exposure. Data are mean ± S.D. values (*n* = 7-8 mice/group). **p < 0.01 compared with corresponding mice exposed to 21% NBO; ^##^*p* < 0.01 compared with corresponding mice exposed to 70% NBO; ††*p* < 0.01/†*p* < 0.05 compared with corresponding mice receiving vehicle.

**Figure 2:**
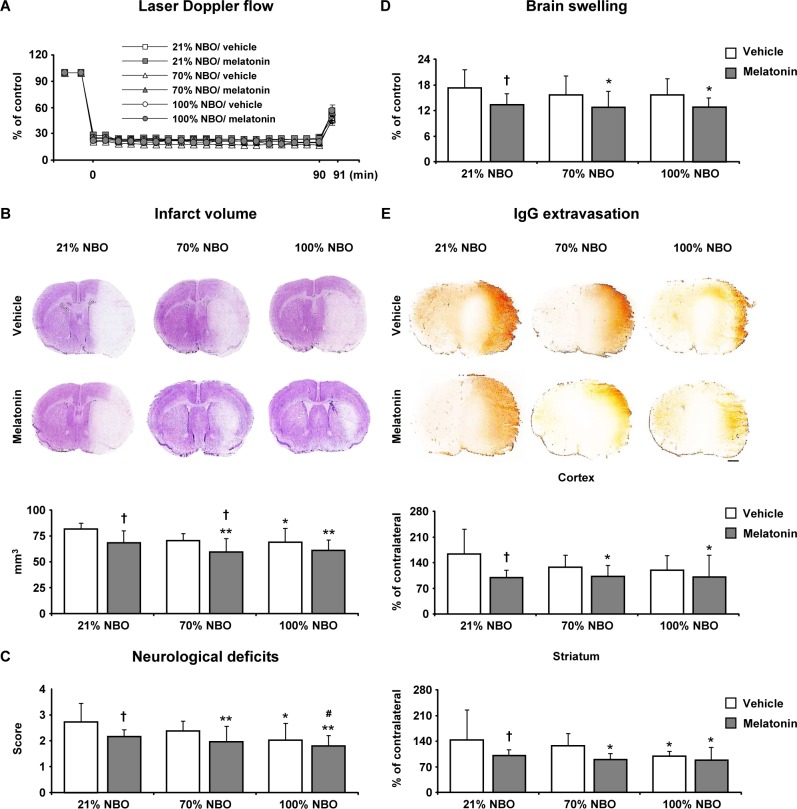
Effects of normobaric oxygen and melatonin exposure on neurological deficits, infarct volume, brain swelling and blood-brain barrier permeability **A.** Laser Doppler flow (LDF) above the core of the MCA territory, **B.** infarct volume, **C.** neurological deficits, **D.** brain swelling and **E.** blood-brain barrier permeability evaluated by serum IgG extravasation in mice submitted to 90 min of intraluminal MCAo, which induces brain infarcts of the striatum and overlying cortex [22]. During the first 90 min of reperfusion, mice had been exposed to 21% (ambient air), 70% and 100% NBO. Vehicle or melatonin (4 mg/kg) was intraperitoneally administered after reperfusion onset, and mice were sacrificed 24 hours later. Note that the absence of synergistic effects of combined NBO and melatonin exposure on neurological deficits, infarct volume, brain swelling and blood-brain barrier permeability. Data are mean ± SD values (*n* = 7-8 mice/group). **p < 0.01/*p < 0.05 compared with corresponding mice exposed to 21% NBO; ^#^*p* < 0.05 compared with corresponding mice exposed to 70% NBO; †*p* < 0.05 compared with corresponding mice receiving vehicle.

To ensure the reproducibility of ischemia, we analyzed LDF recordings above the core of the MCA region. MCAo resulted in a decrease in LDF values to approximately 15% of pre-ischemic level (Figures 1A, 2A). To determine how NBO exposure influenced neuronal injury in the presence and absence of melatonin, we examined mice exposed to 30 min MCAo, which induces disseminate neuronal injury in the striatum that predominantly affects small- to medium-sized interneurons [3, 21]. TUNEL staining revealed that the delivery of NBO alone or melatonin alone decreased neuronal injury (Figure 1B). Importantly, disseminate neuronal injury was more strongly reduced after combined NBO and melatonin delivery than after delivery of either NBO or melatonin (Figure 1B), indicating that NBO and melatonin had synergistic neuroprotective properties.

### Effects of NBO and melatonin on neurological deficits, infarct volume and blood-brain barrier permeability

Studies in mice exposed to 90 min MCAo, which induces brain infarction of the striatum and overlying cortex [22] revealed that NBO alone and melatonin alone reduced infarct volume, neurological deficits and BBB permeability; while melatonin alone, but not NBO alone, decreased brain swelling (Figure 2B–2E). Importantly, the combined NBO and melatonin delivery did not have any additive effects on neurological deficits, infarct volume, brain edema and BBB permeability, neither when 70% nor 100% oxygen was supplied.

### Effects of NBO and melatonin on regional cerebral blood flow

**Figure 3:**
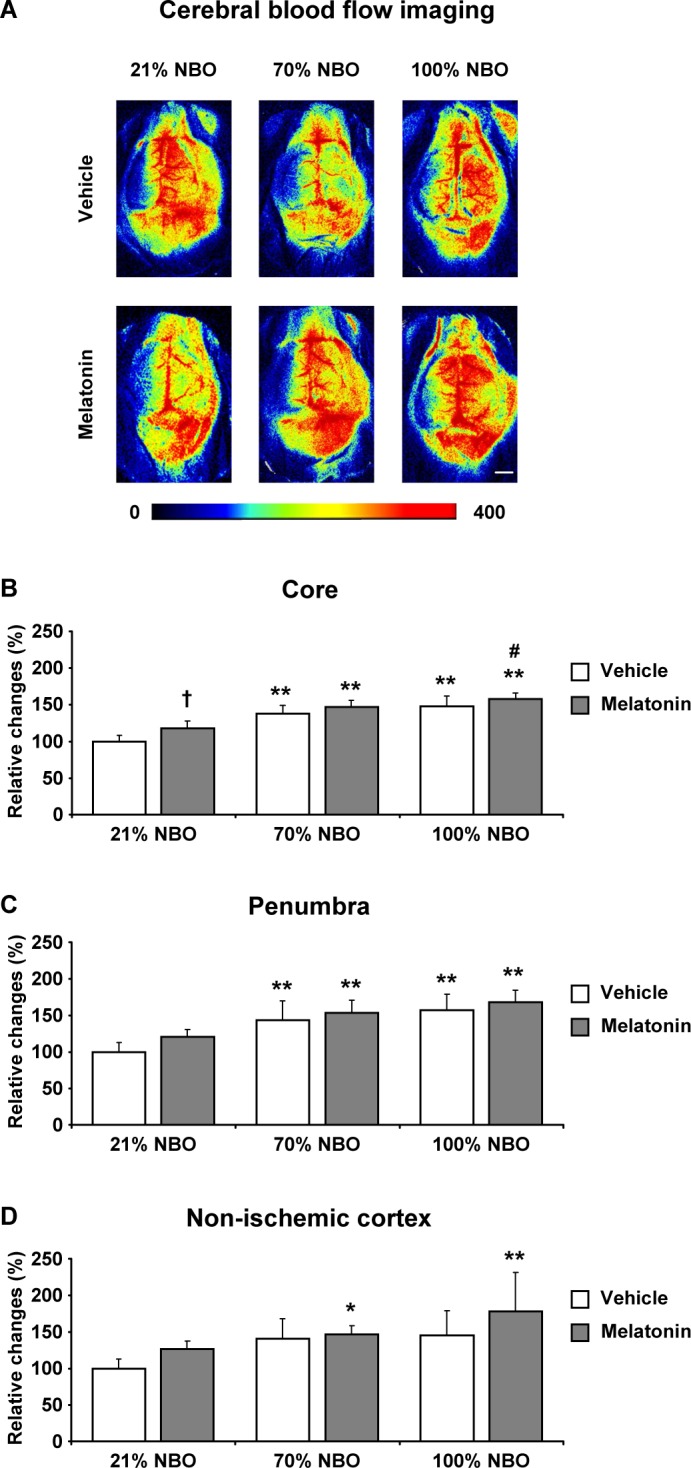
Effects of normobaric oxygen and melatonin on post-ischemic regional cerebral blood flow **A.** Representative examples and **B.**-**D.** semi-quantitative analysis of regional cerebral blood flow (CBF) in the ischemic core (in **B.**), in the ischemic penumbra (in **C.**) and in ipsilateral non-ischemic tissue (in **D.**) evaluated by laser speckle imaging (LSI) in mice submitted to 90 min of intraluminal MCAo. During the initial 90 min of reperfusion, mice had been exposed to 21% (ambient air), 70% and 100% NBO. Vehicle or melatonin (4 mg/kg) was intraperitoneally administered after reperfusion onset, and regional CBF was recorded over 90 min starting 1 min after reperfusion. Regional CBF values (in arbitrary units) were measured in the beginning and end of the recording phase. From these values, relative changes were determined for each region of interest, which were normalized with values determined in mice receiving 21% NBO combined with vehicle (set as 100%). Note that regional CBF, which is markedly increased by NBO above the ischemic core and penumbra **B.**, **C.**, does not further rise in mice exposed to NBO plus melatonin. Data are mean ± SD values (*n* = 4 mice/group). **p < 0.01/*p < 0.05 compared with corresponding mice exposed to 21% NBO; ^#^*p* < 0.05 compared with corresponding mice exposed to 70% NBO; †*p* < 0.05 compared with corresponding mice receiving vehicle.

To evaluate the hemodynamic effects induced by NBO and melatonin, we next analyzed regional CBF in the ischemic core, the ischemic penumbra and ipsilateral non-ischemic tissue by LSI. For this study, we examined mice exposed to 90 min MCAo, which - unlike shorter-lasting ischemia - is followed by secondary hypoperfusion that develops within 90 min after reperfusion onset [23]. Notably, both NBO alone and melatonin alone increased regional CBF above the ischemic core and penumbra (Figure 3A–3D). Thus, regional CBF in mice receiving NBO and melatonin was very similar to mice receiving NBO only.

### Effects of NBO and melatonin on survival-related cell signaling

**Figure 4:**
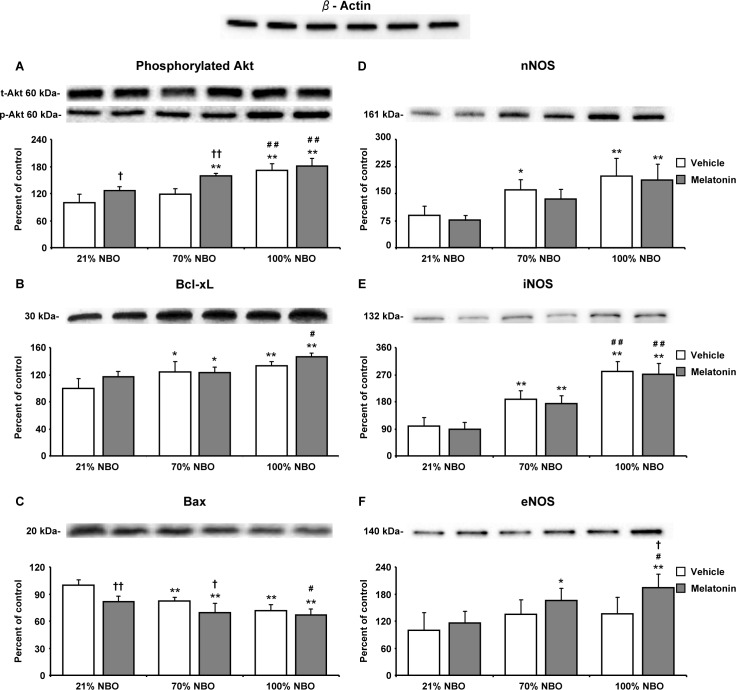
Combination of normobaric oxygen and melatonin re-regulates survival-related proteins, but does not attenuate neuronal and inducible nitric oxide synthases Western blots for **A.** phosphorylated Akt, **B.** anti-apoptotic Bcl-xL, **C.** pro-apoptotic Bax, **D.** neuronal nitric oxide synthase (nNOS), **E.** inducible nitric oxide synthase (iNOS) and **F.** endothelial nitric oxide synthase (eNOS) in ischemic tissue samples obtained from brains of mice submitted to 90 min of intraluminal MCAo. During the first 90 min of reperfusion, mice had been exposed to 21% (ambient air), 70% and 100% NBO. Vehicle or melatonin (4 mg/kg) was intraperitoneally administered after reperfusion onset, and mice were sacrificed 24 hours later. Note that phosphorylated Akt, Bcl-xL, Bax and eNOS are re-regulated by NBO and melatonin, whereas nNOS and iNOS, which are increased by NBO, are not influenced by melatonin. On top of the figure, a representative β-actin blot is shown. Data are mean ± SD values (*n* = 3-5 blots/protein). **p < 0.01/*p < 0.05 compared with corresponding mice exposed to 21% NBO; ^##^*p* < 0.01/^#^*p* < 0.05 compared with corresponding mice exposed to 70% NBO; ††*p* < 0.01/†*p* < 0.05 compared with corresponding mice receiving vehicle.

To characterize the effects of NBO and melatonin on survival-related cell signaling, we analyzed protein abundance in ischemic tissue samples from mice exposed to 90 min MCAo by Western blot. Both NBO alone and melatonin alone increased the levels of phosphorylated (i.e., activated) Akt and Bcl-xL proteins, while the level of total (i.e., phosphorylated and non-phosphorylated) Akt remained unchanged (Figure 4A, 4B). Besides, NBO but not melatonin reduced the level of pro-apoptotic Bax protein (Figure 4C). Interestingly, the levels of phosphorylated Akt and Bcl-xL were highest, while the level of Bax was lowest in mice receiving NBO combined with melatonin (Figure 4A–4C). Levels of nNOS and iNOS, which are both associated with oxidative stress, were dose-dependently increased by NBO (Figure 4D, 4E). Notably, nNOS and iNOS levels were not influenced by melatonin, neither when applied alone, nor in combination with NBO (Figure 4D, 4E). On the other hand, eNOS level was neither influenced by NBO alone, nor melatonin alone, but was significantly increased after combined NBO and melatonin delivery (Figure 4F).

## DISCUSSION

Hopes have emerged from hitherto performed studies that NBO and melatonin are promising approaches for the treatment of acute-ischemic stroke. However, at least in theoretical aspect, hyperoxia has been criticized and remains controversial owing to the lack of comprehensive studies focusing on reperfusion injury, and underlying mechanism, and the risk of enhanced over-production of ROS. In this context, we have examined the effect of NBO treatment alone or in combination with melatonin after focal cerebral ischemia. For this aim, a total of three sets of experiments were designed. Animals were treated with 21, 70 and 100% NBO in combination with melatonin during reperfusion. In the first set of experiments, animals were submitted to 30 minutes of focal cerebral ischemia and 72 hours of reperfusion, which causes disseminated apoptotic cell death in the striatum, for the evaluation of DNA fragmentation. In the second and third set of experiments, animals were submitted to 90 minutes of focal cerebral ischemia and 24 hours of reperfusion for the evaluation of i) real-time semi-quantitative cerebral microcirculation by using LSI in the ischemic- cortex, penumbra and non-ischemic cortex; ii) infarct volume, brain swelling and neurological deficit score; iii) IgG extravasation indicating BBB permeability; and iv) Western blot analysis including iNOS, eNOS (eNOS), nNOS (nNOS), anti-apoptotic Bcl-xL, pro-apoptotic key factor Bax, and survival kinase AKT, which play significant roles in the pathology of ischemic brain injury.

Herein, we show that combined NBO and melatonin delivery synergistically reduces disseminate neuronal injury after mild focal cerebral ischemia induced by 30 min MCAo. Both NBO and particularly melatonin alone reduced neuronal injury, neurological deficits, infarct volume and BBB permeability, and increased post-ischemic cerebral blood flow evaluated by LSI after 90 min of MCAo. Levels of phosphorylated Akt, anti-apoptotic Bcl-xL, pro-apoptotic Bax and endothelial NO synthase were re-regulated after combined NBO and melatonin delivery, whereas levels of neuronal and inducible NO synthase, which were increased by NBO, were not influenced by melatonin.

Tissue re-oxygenation is critical for the survival of ischemic tissue, and NBO is highly efficient in reversing interstitial pO_2_ deficits. Thus, NBO administered during focal cerebral ischemia was able to maintain penumbral interstitial pO_2_ close to the pre-ischemic values, whereas NBO delivery during reperfusion still doubled penumbral pO_2_ that was otherwise reduced to ≈30% [5]. Yet, the therapeutic utility of NBO remained controversial, since NBO delivered during ischemia [5, 7, 8] but not reperfusion [5, 9], was found to decrease infarct volume and improve neurological deficits. The lack of therapeutical effects has been attributed to increased ROS formation triggering lipid peroxidation, membrane damage and neuronal death [10]. In our present study, we evaluated the favorable or unfavorable effect of NBO treatment on reperfusion injury thoroughly by using two different MCAo models (30 or 90 minute-durations). Notably, we have not observed any unfavorable effects of NBO treatment on the reperfusion injury as compared with normoxia, which was 21% of O_2_ inhalation. It is noteworthy that tissue survival was improved progressively when animals were treated with increased concentration of NBO. However, melatonin treatment alone was more efficacious as compared with NBO treatment at all concentrations studied. Furthermore, melatonin potentiated the protective effect of NBO treatment significantly, evaluated by infarct volume, brain swelling, neurological deficit score and DNA fragmentation.

In the human vascular system, it was revealed that 100% O_2_ treatment causes vasoconstriction and reduces CBF around 20% in healthy subjects [24]. However, in a pre-clinical stroke study, it was shown that hyperoxia preserves CBF in both core and penumbra, when NBO was applied during focal cerebral ischemia [7]. In the case of melatonin, diverse effects of melatonin's MT1 and MT2 receptors on CBF have been reported [25]. However, in our recent study, we observed that melatonin improves CBF under ischemic stroke conditions by inhibiting ECE-1 [14, 17, 20] Furthermore, we revealed that the beneficial effect of melatonin on CBF was independent from MT1 and MT2 receptors [14]. However in these studies CBF analysis were conducted by LDF using a flexible optic probe, which was attached to the intact skull overlying the MCA territory. LDF does not provide precise measurements of absolute regional CBF values; it provides noninvasive, instantaneous, and continuous measurement of microcirculatory blood flow in a small tissue sample [26]. Therefore, it remained a matter of further research using a more precise CBF analysis method that allows spatial resolution. Therefore, in the present study, we used LSI for the analysis of CBF on the ischemic core, penumbra and non-ischemic brain tissue. We have observed that both melatonin and NBO treatment improved CBF significantly. In addition, the effects of melatonin and NBO treatment on the BBB permeability were evaluated by IgG extravasation analysis. Consistent with brain swelling, it was observed that both NBO and especially melatonin reduced BBB permeability significantly.

In line with the observation that combined NBO and melatonin delivery reduced disseminate neuronal injury, levels of phosphorylated Akt, anti-apoptotic Bcl-xL, pro-apoptotic Bax and eNOS were re-regulated upon combined NBO and melatonin delivery. Thus, levels of phosphorylated Akt, Bcl-xL and eNOS were increased, whereas the level of Bax was reduced, as revealed in Western blots. On the other hand, nNOS and iNOS activities were increased by NBO, but not influenced by melatonin. It has been previously reported that Bcl-xL is upregulated and Bax is downregulated by NBO after intraluminal MCAo in rats [27]. Upregulation of phosphorylated Akt, Bcl-xL and eNOS and downregulation of Bax, nNOS and iNOS by melatonin have been described after intraluminal MCAo in mice [14, 16, 17] or in rats [19]. Production of ROS, nitric oxide, nNOS and iNOS contribute to lipid peroxidation and cellular energy failure [28].

In conclusion, both NBO and melatonin synergistically improved CBF significantly in the ischemic- core and penumbra analyzed by real-time laser speckle imaging, which was associated with reduced IgG extravasation, DNA fragmentation, infarct volume, brain swelling and neurological scores which was associated with decreased activity of pro-apoptotic protein Bax and increased activities of NOSs, anti-apoptotic Bcl-xL and survival kinase Akt. Our study suggests the utility of NBO as add-on treatment to free radical scavenging drugs.

## MATERIALS AND METHODS

### Ethics statement

This study has been conducted in accordance with the ethical standards and according to the Declaration of Helsinki and according to national and international guidelines and has been approved by the Ethics Committee of Istanbul Medipol University.

### Experimental groups

Experiments were performed using male Balb/c mice (22-25 g). A total of three sets of mice were examined, which were randomly treated with 21%, 70% or 100% NBO during reperfusion with intraperitoneal delivery of vehicle (50 μl isotonic saline/5% ethanol) or melatonin (4 mg/kg, dissolved in 0.9% isotonic saline/5% ethanol) at the moment of reperfusion onset.

In the first set, mice were exposed to 30 min of focal cerebral ischemia followed by 72 hours reperfusion. These mice were used for the evaluation of disseminate ischemic injury in the striatum (*n* = 7-8/per group). The second set of mice was exposed to 90 min of focal cerebral ischemia followed by 24 hours reperfusion for the analysis of infarct volume, IgG extravasation, brain swelling and cell signaling pathways (*n* = 7-8/per group). The third set was exposed to 90 min of focal cerebral ischemia followed by real-time evaluation of CBF by LSI during the first 90 min after reperfusion onset (*n* = 4/per group).

### Animal surgery

Mice were anesthetized with 1% isofluorane (30% O_2_, reminder N_2_O) and rectal temperature was controlled between 36.5 and 37.0°C using a feedback-controlled heating system. During the experiments, CBF was monitored via laser Doppler flowmetry (LDF) using a flexible 0.5 mm fiber optic probe (Perimed, Sweden) which was attached with tissue adhesive to the intact skull overlying the MCA territory (2 mm posterior and 6 mm lateral from bregma). Focal cerebral ischemia was induced using an intraluminal filament technique [16]. Briefly, after a midline neck incision the left common and external carotid arteries were isolated and ligated. A microvascular clip (FE691; Aesculap) was temporarily placed on the internal carotid artery. A 8-0 nylon monofilament (Ethilon; Ethicon, Norderstedt, Germany) coated with silicon resin (Xantopren; Bayer Dental, Osaka, Japon; diameter of the coated suture 180 to 190 μm) was inserted through a small incision into the common carotid artery and advanced 9 mm distal to the carotid bifurcation for MCAo. Reperfusion was initiated 30 or 90 min after onset of ischemia by gentle monofilament removal.

One minute after reperfusion, anesthesia was terminated, and mice were placed into an oxygen chamber (R = 25cm), in which they were exposed to ambient air (21%) or normobaric hyperoxia (70% or 100% O_2;_ remainder N_2_) over 90 minutes. At the same moment, 50 μl of either vehicle (0.9% sodium chloride/5% ethanol) or melatonin (4 mg/kg, dissolved in 0.9% sodium chloride/5% ethanol) were intraperitoneally administered. During NBO exposure, internal pressure of the chamber was continuously observed with a monometer and the internal oxygen concentration was controlled with an oxygen sensor (MX-300; Teledyne Analytical Instruments). Thereafter, mice were placed back into their home cages.

In mice exposed to 90 min MCAo, neurological deficits were evaluated 24 hours after MCAo using the following 5-point score: 0 = normal function; 1 = flexion of torso and of the contralateral forelimb upon lifting of the animal by the tail; 2 = circling to the contralateral side but normal posture at rest; 3 = reclination to the contralateral side at rest; 4 = absence of spontaneous motor activity. At 24 hours (for 90 min MCAo) or 72 hours (for 30 min MCAo) after reperfusion, mice were sacrificed under deep anesthesia (4% isofluorane with 30% O_2_, remainder N_2_O). Brains were removed, frozen on dry ice and cut on a cryostat into coronal 18-μm sections, which were subsequently used for the analysis of disseminate neuronal injury, infarct volume, serum IgG extravasation and brain swelling. From the same mice, tissue samples obtained from the striatum ipsilateral and contralateral to the stroke were pooled for Western blots.

### Laser speckle imaging

Mice were anesthetized with 1% isofluorane (30% O_2_, remainder N_2_O) and placed in a stereotactic frame (World Precision Instruments, Berlin, Germany). Throughout the experimental procedure, rectal temperature was maintained between 36.5 and 37.0°C using a feedback-controlled heating system. A midline incision was made in the scalp and the skull surface was cleaned with sterile normal saline. Both ipsilateral and contralateral skull bones were carefully opened using a high speed dental drill (Marothon-3; Korea), leaving the underlying dura mater intact throughout the experiment. Mice were then exposed to 90 min MCAo as described above. Starting 1 min after reperfusion, real-time CBF changes were recorded by means of a CCD camera that was placed roughly 10 cm above the brain using a Pericam PSI System (Perimed). The penetration depth of the laser (785 nm) is approximately 500 μm below the brain surface. Raw speckle images were taken at 2-second-intervals with a spatial image resolution of 20 μm. To evaluate the CBF changes in the ischemic core, ischemic penumbra and ischemia-remote cortex, regions of interest (ROI) covering 1.0 mm × 5.5 mm (in lateral and rostrocaudal direction, respectively) were defined 0.5, 1.5 and 2.5 mm lateral and 0.5 mm posterior to the bregma, in which mean CBF was calculated using a blood perfusion imaging software (PIMSoft; Perimed) [23, 29]. Regional CBF was recorded throughout the 90 min observation period. From the measurements obtained, relative CBF changes (in %) at the end of the observation period compared to the beginning of the observation period were calculated, which were normalized with values obtained in the vehicle/21% oxygen group, which were set at 100%.

### Analysis of neuronal injury

For the evaluation of neuronal injury, coronal brain sections at the level of the bregma from mice exposed to 30 min MCAo were fixed with 4% paraformaldehyde (PFA)/0.1 M phosphate buffered saline (PBS) and were labeled using a TUNEL kit (In Situ Cell Death Detection Kit; Roche, Switzerland). Sections were counterstained with 4′,6-diamidino-2-phenylindole (DAPI). Stainings were analyzed by quantifying DNA-fragmented cells (which in 30 min MCAo are equivalent to neurons) in twelve adjacent ROI in the striatum, each measuring 62,500 μm^2^, under a confocal Zeiss LSM 780 microscope (Carl Zeiss, Jena, Germany).

### Analysis of infarct volume and brain swelling

For the evaluation of infarct volume and brain swelling, coronal brain sections were collected at four equidistant brain levels, 2 mm apart, from mice exposed to 90 min MCAo, which were stained with cresyl violet according to a standard protocol. Within the sections, the border between infarcted and non-infarcted tissues was outlined using an image analysis system (Image J; National Institute of Health, Bethesda, MD, USA), and infarct area was assessed by subtracting the area of the non-infarcted ipsilateral hemisphere from that of the contralateral side. Infarct volume was calculated by integration of these infarct areas. Edema was calculated as the volume difference between the ischemic and the non-ischemic hemisphere and expressed as percentage of the volume of the non-ischemic hemisphere.

### Analysis of serum IgG extravasation

With gentle stirring, brain sections from the bregma level of mice exposed to 90 min MCAo were rinsed for 10 min at room temperature in 0.1 M PBS to remove intravascular IgG, and were fixed in 4% PFA [30]. Following the blocking of endogenous peroxidase with methanol/0.3% H_2_O_2_ and immersion in 0.1 M PBS containing 5% bovine serum albumin (BSA) and normal swine serum (1:1000), sections were incubated for 1 h in biotinylated goat anti-mouse IgG (sc-2013; Santa Cruz Biotechnology), and stained with an avidin peroxidase kit (Vectastain Elite; Vector Labs) and diaminobenzidine (Sigma). For reasons of data comparability, all sections were processed in parallel. Sections were scanned and IgG extravasation in the ischemic striatum and cortex was densitometrically analyzed. For correction of background staining, optical densities in corresponding contralateral non-ischemic tissue were subtracted from those in the ischemic tissue.

### Western blot

For Western blot analysis, brain tissue samples were harvested from the ischemic striatum of mice exposed to 90 min MCAo. Tissue samples belonging to the same group were pooled, homogenized, sonicated, and treated with protease inhibitor cocktail and phosphatase inhibitor cocktail. Total protein content was evaluated using Qubit 2.0 Fluorometer according to the manufacturer's protocol (Invitrogen, Life Technologies Corporation, Carlsbad, CA, USA). Equal amounts of protein (20 μg) were size-fractionated using any-kD Mini-Protean TGX gel electrophoresis and then transferred to a nitrocellulose membrane using the Trans-Blot TurboTransfer System (Bio-Rad, Life Sciences Research). Thereafter, membranes were blocked in 5% nonfat milk in 50 mMol Tris-buffered saline containing 0.1% Tween (TBS-T; blocking solution) for 1 hour at room temperature, washed in 50 mMol TBS-T, and incubated overnight with polyclonal rabbit anti-total (detecting both the phosphorylated and unphosphorylated form) Akt (9272; Cell Signaling), polyclonal rabbit anti-phosphorylated Akt (9271; Cell Signaling), monoclonal rabbit anti-Bcl-xL (2764; Cell Signaling), rabbit polyclonal anti-Bax (2772; Cell Signaling), rabbit monoclonal anti-nNOS (Ab76067; Abcam), rabbit polyclonal anti-iNOS (sc-650; Santa Cruz Biotechnology) and rabbit polyclonal anti-eNOS (Ab66127; Abcam) antibody (all diluted 1:000). The next day, membranes were washed with 50 mM TBS-T and incubated with horseradish peroxidase-conjugated goat-anti rabbit (31460; Thermo Scientific) antibody (diluted 1:2500) for 1 hour at room temperature. Blots were performed at least three times. Protein loading was controlled by stripping and reprobing with polyclonal rabbit anti-β-actin antibody (4967; Cell Signaling Technology). Blots were developed using Clarity Western ECL Substrate kit (Bio-Rad; Life Sciences Research) and visualized using the ChemiDoc MP System (Bio-Rad; Life Sciences Research). Protein levels were analyzed densitometrically using the ImageJ program, corrected with values determined on β-actin blots and expressed as relative values compared with vehicle treated mice exposed to 21% NBO.

### Statistics

Data were evaluated by one-way ANOVA followed by LSD tests. Data are presented as mean ± S.D. values. Throughout the study, p values < 0.05 were considered significant.

## References

[1] Furlan AJ (2015). Endovascular therapy for stroke--it's about time. The New England journal of medicine.

[2] Hata R, Maeda K, Hermann D, Mies G, Hossmann KA (2000). Evolution of brain infarction after transient focal cerebral ischemia in mice. Journal of cerebral blood flow and metabolism : official journal of the International Society of Cerebral Blood Flow and Metabolism.

[3] Hermann DM, Kilic E, Hata R, Hossmann KA, Mies G (2001). Relationship between metabolic dysfunctions, gene responses and delayed cell death after mild focal cerebral ischemia in mice. Neuroscience.

[4] Dirnagl U, Iadecola C, Moskowitz MA (1999). Pathobiology of ischaemic stroke: an integrated view. Trends in neurosciences.

[5] Liu S, Liu W, Ding W, Miyake M, Rosenberg GA, Liu KJ (2006). Electron paramagnetic resonance-guided normobaric hyperoxia treatment protects the brain by maintaining penumbral oxygenation in a rat model of transient focal cerebral ischemia. Journal of cerebral blood flow and metabolism : official journal of the International Society of Cerebral Blood Flow and Metabolism.

[6] Esposito E, Mandeville ET, Hayakawa K, Singhal AB, Lo EH (2013). Effects of normobaric oxygen on the progression of focal cerebral ischemia in rats. Experimental neurology.

[7] Shin HK, Dunn AK, Jones PB, Boas DA, Lo EH, Moskowitz MA, Ayata C (2007). Normobaric hyperoxia improves cerebral blood flow and oxygenation, and inhibits peri-infarct depolarizations in experimental focal ischaemia. Brain : a journal of neurology.

[8] Singhal AB, Wang X, Sumii T, Mori T, Lo EH (2002). Effects of normobaric hyperoxia in a rat model of focal cerebral ischemia-reperfusion. Journal of cerebral blood flow and metabolism : official journal of the International Society of Cerebral Blood Flow and Metabolism.

[9] Flynn EP, Auer RN (2002). Eubaric hyperoxemia and experimental cerebral infarction. Annals of neurology.

[10] Reiter RJ, Tan DX, Leon J, Kilic U, Kilic E (2005). When melatonin gets on your nerves: its beneficial actions in experimental models of stroke. Experimental biology and medicine.

[11] Galano A, Tan DX, Reiter RJ (2011). Melatonin as a naturalally against oxidative stress: a physicochemical examination. Journal of pineal research.

[12] Reiter RJ, Tan DX, Fuentes-Broto L (2010). Melatonin: a multitasking molecule. Progress in brain research.

[13] Tai SH, Chen HY, Lee EJ, Chen TY, Lin HW, Hung YC, Huang SY, Chen YH, Lee WT, Wu TS (2010). Melatonin inhibits postischemic matrix metalloproteinase-9 (MMP-9) activation via dual modulation of plasminogen/plasmin system and endogenous MMP inhibitor in mice subjected to transient focal cerebral ischemia. Journal of pineal research.

[14] Kilic U, Yilmaz B, Ugur M, Yuksel A, Reiter RJ, Hermann DM, Kilic E (2012). Evidence that membrane-bound G protein-coupled melatonin receptors MT1 and MT2 are not involved in the neuroprotective effects of melatonin in focal cerebral ischemia. Journal of pineal research.

[15] Kilic E, Kilic U, Yulug B, Hermann DM, Reiter RJ (2004). Melatonin reduces disseminate neuronal death after mild focal ischemia in mice via inhibition of caspase-3 and is suitable as an add-on treatment to tissue-plasminogen activator. Journal of pineal research.

[16] Kilic E, Kilic U, Soliz J, Bassetti CL, Gassmann M, Hermann DM (2005). Brain-derived erythropoietin protects from focal cerebral ischemia by dual activation of ERK-1/-2 and Akt pathways. FASEB journal : official publication of the Federation of American Societies for Experimental Biology.

[17] Kilic E, Kilic U, Reiter RJ, Bassetti CL, Hermann DM (2005). Tissue-plasminogen activator-induced ischemic brain injury is reversed by melatonin: role of iNOS and Akt. Journal of pineal research.

[18] Pei Z, Fung PC, Cheung RT (2003). Melatonin reduces nitric oxide level during ischemia but not blood-brain barrier breakdown during reperfusion in a rat middle cerebral artery occlusion stroke model. Journal of pineal research.

[19] Koh PO (2008). Melatonin regulates nitric oxide synthase expression in ischemic brain injury. The Journal of veterinary medical science / the Japanese Society of Veterinary Science.

[20] Kilic E, Ozdemir YG, Bolay H, Kelestimur H, Dalkara T (1999). Pinealectomy aggravates and melatonin administration attenuates brain damage in focal ischemia. Journal of cerebral blood flow and metabolism: official journal of the International Society of Cerebral Blood Flow and Metabolism.

[21] Bacigaluppi M, Pluchino S, Peruzzotti-Jametti L, Kilic E, Kilic U, Salani G, Brambilla E, West MJ, Comi G, Martino G, Hermann DM (2009). Delayed post-ischaemic neuroprotection following systemic neural stem cell transplantation involves multiple mechanisms. Brain : a journal of neurology.

[22] Spudich A, Kilic E, Xing H, Kilic U, Rentsch KM, Wunderli-Allenspach H, Bassetti CL, Hermann DM (2006). Inhibition of multidrug resistance transporter-1 facilitates neuroprotective therapies after focal cerebral ischemia. Nature neuroscience.

[23] Kilic E, Bahr M, Hermann DM (2001). Effects of recombinant tissue plasminogen activator after intraluminal thread occlusion in mice: role of hemodynamic alterations. Stroke; a journal of cerebral circulation.

[24] Watson NA, Beards SC, Altaf N, Kassner A, Jackson A (2000). The effect of hyperoxia on cerebral blood flow: a study in healthy volunteers using magnetic resonance phase-contrast angiography. European journal of anaesthesiology.

[25] Doolen S, Krause DN, Dubocovich ML, Duckles SP (1998). Melatonin mediates two distinct responses in vascular smooth muscle. European journal of pharmacology.

[26] Dirnagl U, Kaplan B, Jacewicz M, Pulsinelli W (1989). Continuous measurement of cerebral cortical blood flow by laser-Doppler flowmetry in a rat stroke model. Journal of cerebral blood flow and metabolism : official journal of the International Society of Cerebral Blood Flow and Metabolism.

[27] Geng X, Parmar S, Li X, Peng C, Ji X, Chakraborty T, Li WA, Du H, Tan X, Ling F, Guthikonda M, Rafols JA, Ding Y (2013). Reduced apoptosis by combining normobaric oxygenation with ethanol in transient ischemic stroke. Brain research.

[28] Volpe M, Cosentino F (2000). Abnormalities of endothelial function in the pathogenesis of stroke: the importance of endothelin. Journal of cardiovascular pharmacology.

[29] Hori M, Nakamachi T, Shibato J, Rakwal, Seiji S, Numazawa S (2015). Unraveling the Specific Ischemic Core and Penumbra Transcriptome in the Permanent Middle Cerebral Artery Occlusion Mouse Model Brain Treated with the Neuropeptide PACAP38. Microarrays.

[30] Kilic E, Kilic U, Wang Y, Bassetti CL, Marti HH, Hermann DM (2006). The phosphatidylinositol-3 kinase/Akt pathway mediates VEGF's neuroprotective activity and induces blood brain barrier permeability after focal cerebral ischemia. FASEB J.

